# Intracellular Recording of Cardiomyocytes by Integrated Electrical Signal Recording and Electrical Pulse Regulating System

**DOI:** 10.3389/fbioe.2021.799312

**Published:** 2021-12-15

**Authors:** Zhengjie Liu, Dongxin Xu, Jiaru Fang, Qijian Xia, Wenxi Zhong, Hongbo Li, Zhanyun Huang, Nan Cao, Xingxing Liu, Hui-Jiuan Chen, Ning Hu

**Affiliations:** ^1^ State Key Laboratory of Optoelectronic Materials and Technologies, Guangdong Province Key Laboratory of Display Material and Technology, School of Electronics and Information Technology, The First Affiliated Hospital of Sun Yat-sen University, Sun Yat-sen University, Guangzhou, China; ^2^ Laboratory Teaching Center of Electronics and Information Technology, Sun Yat-sen University, Guangzhou, China; ^3^ Zhongshan School of Medicine, Sun Yat-sen University, Guangzhou, China; ^4^ State Key Laboratory of Transducer Technology, Chinese Academy of Sciences, Shanghai, China

**Keywords:** cardiomyocytes, intracellular recording, nanopatterned microelectrode array, electroporation, electrophysiological recording

## Abstract

The electrophysiological signal can reflect the basic activity of cardiomyocytes, which is often used to study the working mechanism of heart. Intracellular recording is a powerful technique for studying transmembrane potential, proving a favorable strategy for electrophysiological research. To obtain high-quality and high-throughput intracellular electrical signals, an integrated electrical signal recording and electrical pulse regulating system based on nanopatterned microelectrode array (NPMEA) is developed in this work. Due to the large impedance of the electrode, a high-input impedance preamplifier is required. The high-frequency noise of the circuit and the baseline drift of the sensor are suppressed by a band-pass filter. After amplifying the signal, the data acquisition card (DAQ) is used to collect the signal. Meanwhile, the DAQ is utilized to generate pulses, achieving the electroporation of cells by NPMEA. Each channel uses a voltage follower to improve the pulse driving ability and isolates each electrode. The corresponding recording control software based on LabVIEW is developed to control the DAQ to collect, display and record electrical signals, and generate pulses. This integrated system can achieve high-throughput detection of intracellular electrical signals and provide a reliable recording tool for cell electro-physiological investigation.

## 1 Introduction

Cardiovascular disease is currently the main cause of death in the world, seriously threatening human health (Lin et al., 2021; Mozaffarian et al., 2016). In order to prevent and treat cardiovascular diseases, pathologists have carried out more in-depth research on the working mechanism of the heart. Since the electrophysiological activity of the heart helps to understand the working mechanism, it has been extensively studied (Watanabe et al., 2016). The biological models established for the electrophysiological research include living animals and cells (Yeo et al., 2017). Living animal models are often used in disease research because their cardiovascular systems are highly similar to humans (Zhang et al., 2019; Zhu et al., 2019). However, living animal models are limited to difficult operation, costly feeding and lack of conditions for large-scale research (Fermini and Fossa, 2003; Li et al., 2017). Recently, a number of cell-based technologies and equipment have been developed because of their high throughput and low cost (Crook and Kobayashi, 2008; Du et al., 2016). Among which, the electrical signal recording platform constructed with cardiomyocytes as sensitive unit has become one of the important methods for electrophysiological research. The patch clamp platform as the gold standard technology for studying cell electrophysiological signals can acquire abundant action potential information with high resolution (Shuvaev et al., 2016; Y. Zhao et al., 2008). However, the low throughput, cumbersome operation, high demanding experimental conditions, and invasive detection make it difficult to carry out long-term researches (Black et al., 2018; Sun et al., 2020). With the development of micro-nano technology, high-throughput, non-invasive microelectrode arrays (MEA) are widely used to detect the electrophysiological activity of cells (Stett et al., 2003; Zwartsen et al., 2019). The electrodes of MEA are generally planar, and the detected signals are extracellular signals with low amplitudes. However, these low-quality extracellular signals make it difficult to reflect the true characteristics of intracellular action potentials. Gaps between planar electrodes and cells reduce the signal quality, so MEA combined with three-dimensional (3D) structure have been developed to enhance the cell-electrode coupling, such as spine-shaped (Hai et al., 2009), volcano-shaped (Desbiolles et al., 2019), mushroom-shaped (Hai and Spira, 2012). Some electrodes with 3D structure can achieve spontaneous perforation (Staufer et al., 2019), but most of them need to improve the efficiency of perforation through various aided perforation techniques, including electroporation (Xie et al., 2012; Lin et al., 2014; Abbott et al., 2017; Edwards et al., 2018), chemical modification (Duan et al., 2012; Fu et al., 2014; Yunlong Zhao et al., 2019), mechanical forces (Xu et al., 2014; Dipalo, McGuire, et al., 2018), and plasmonic optoporation (Dipalo et al., 2017; Dipalo, Melle, et al., 2018). In these methods, the chemical modification is complex, the mechanical force has low efficiency, and plasmonic optoporation requires complicated and high-cost equipment. Therefore, electroporation has become a common method.

In order to achieve cell electroporation and cell electrical signal recording, a system combined with function generators and commercial recording instrument has been used to record intracellular and extracellular action potentials (Reberšek, 2017; Zhang et al., 2020; D. Xu, Fang, et al., 2021). However, the function generator for electroporation requires a large volume and complicated operation. To better integrate stimulation and recording functions, many researchers have built their own customized equipment. The use of VLSI to realize CMOS systems with measurement and simulation functions has high integration and performance, but the CMOS manufacturing process is complex and cannot be copied quickly and cheaply in other laboratories (Pancrazio et al., 1998). Therefore, for the ever-increasing need for cell electrophysiological testing, it is very necessary to establish a high-integration and low-cost system that integrates cell electrical signal recording and electrical pulse regulating.

**FIGURE 1:**
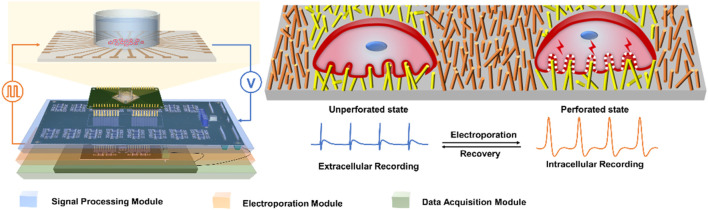
Schematic of integrated electrical signal recording and electrical pulse regulating system based on NPMEA. The multifunctional recording and regulating system shows that the hardware module connected to a NPMEA sensor. The cell on the left is in an unperforated state, the electrode records extracellular electrical signals. The cell on the right is in a perforated state, and the electrode records intracellular electrical signals. A reversible electroporation is applied to the cell that converts the electrode from extracellular (blue) to intracellular recording (orange).

In this work, we have developed an integrated electrical signal recording and electrical pulse regulating system for intracellular recording (Figure 1). In order to verify intracellular electrical signal recording function, the neonatal rat cardiomyocytes are electroporated through the electroporation module. Due to the use of a high aspect ratio nano-patterned microelectrode array (NPMEA), the electrode can form a tight coupling with cardiomyocytes, so the cells can be electroporated by applying low-voltage (∼3 V) pulses. The action potentials of cardiomyocytes recorded by this system are significantly enhanced in amplitude (∼2 mV) and signal-to-noise ratio (SNR) (∼40 dB). In the case of minimal damage to the primary cultured rat cardiomyocytes, high-quality and long-term intracellular recording can be achieved. In addition, the electroporation function of the system can adjust the amplitude, frequency, pulse width and duration of the pulse, a reasonable set of pulse parameters may be capable of forming a reversible electroporation of cardiomyocytes. This integrated system can be used as a practical research platform for electrophysiology in the biomedical field.

## 2 Experimental Methods

### 2.1 Principle of Electrophysiological Signal Detection

**FIGURE 2:**
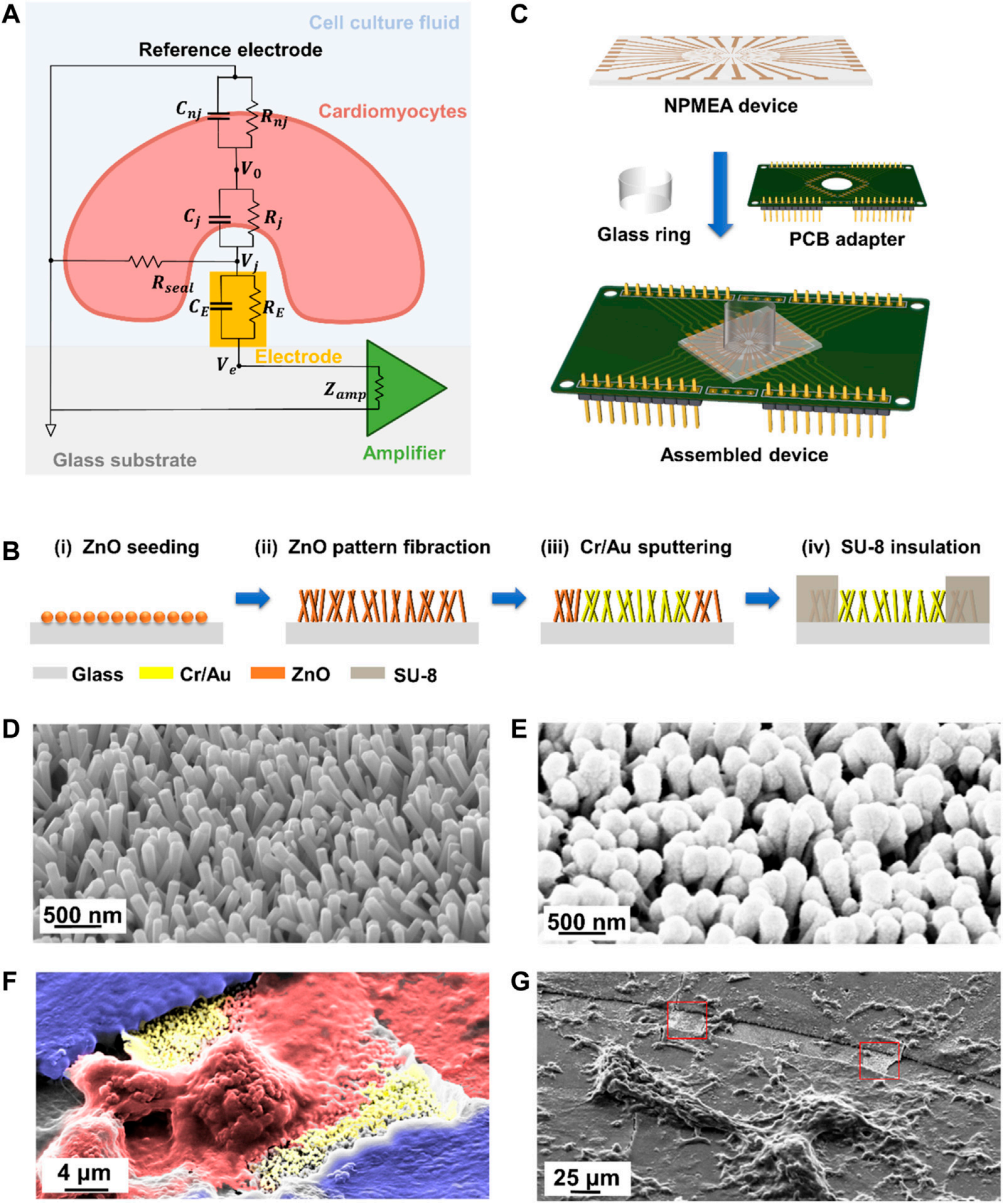
NPMEA for biosensing of cardiomyocytes. **(A)** The equivalent circuit between cardiomyocytes and nanoelectrodes. The cardiomyocyte (light red) resides on a sensing electrode (orange) integrated in the culture substrate (grey). Cell culture medium (light blue) fill the gap between the cell membrane and the electrode-substrate. **(B)** Fabrication process flow to fabricate NPMEA. (i) Sputter ZnO seed layer on the glass substrate. (ii) Synthesis of ZnO nanopatterns based on hydrothermal reaction. (iii) Form a metal layer on the nano pattern by photolithography and sputtering. (iv) Pattern SU-8 layer to the non-electrode area for insulation by photolithography. **(C)** Fix the nanopatterned microelectrode array device to the PCB with PDMS, connect the matching pad of the device and the PCB with conductive silver glue, and then glue the glass ring to the center of the device with PDMS. **(D)** Scanning electron microscope (SEM) image of the ZnO pattern by the hydrothermal growth, corresponding to 2B(ii). **(E)** SEM image of the ZnO pattern after Cr/Au sputtering, corresponding to 2B(iii). **(F)** Colored SEM image of cardiomyocyte (red) on nanopatterned electrodes (yellow). **(G)** SEM image of NPMEA cultured with cardiomyocytes. Exposed nanopatterned microelectrodes are shown in the red box.

In order to visually describe the NPMEA intracellular recording, the equivalent circuit between cardiomyocytes and microelectrodes is shown in the Figure 2A (Spira and Hai, 2013; Shmoel et al., 2016), cardiomyocytes are divided into: the junctional membrane facing the microelectrodes ( ${\text{R}}_{j}$ and ${\text{C}}_{j}$ form the junction impedance ${\text{Z}}_{j}$ ) and the non-connected membrane ( ${\text{C}}_{nj}$ and ${\text{R}}_{nj}$ are non-junction capacitance and resistance) facing the culture medium and the substrate. Gaps formed between the cardiomyocytes and the microelectrodes will generate a resistance called the sealing resistance ( ${\text{R}}_{\text{seal}}$ ). Electrode impedance $Z_{e}$ is composed of electrode resistance and capacitance ( ${\text{R}}_{e}$ and ${\text{C}}_{e}$ respectively). $Z_{amp}$ is the impedance of the amplifier. ${\text{V}}_{0}$ is the action potential spontaneously produced by cardiomyocytes. ${\text{V}}_{\text{e}}$ is the voltage of the electrode, which is the electrical signal to be collected. Define the ratio of ${\text{V}}_{\text{e}}$ to ${\text{V}}_{0}$ as η, which means the cell-electrode coupling efficiency, and it can be written as (D. Xu, Mo, et al., 2021):

$$\mathrm{\eta =}\frac{V_{e}}{V_{0}}=\frac{R_{\mathrm{seal}}\times Z_{\mathrm{amp}}}{\;Z_{j}\times R_{\mathrm{seal}}+Z_{\mathrm{amp}}+Z_{e}+R_{\mathrm{seal}}\times \left(Z_{\mathrm{amp}}+Z_{e}\right)}$$ (1)

From Eq. 1, coupling efficiency η is inversely proportional to junction impedance $Z_{j}$ . Before electroporation, the electrode is located outside the cell membrane, and the junction impedance $Z_{j}$ is high. When electroporation is performed to induce nanopores on the cell membrane, the junction impedance $Z_{j}$ decreases significantly, which improves the quality of the intracellular signal.

### 2.2 Sensor Fabrication

As illustrated in Figure 2B, NPMEA is fabricated by a simplified strategy combined with hydrothermal growth and standard microfabrication. In the first step, glass with a thickness of 1 mm is selected as the substrate. After ultrasonic cleaning, the substrate is spin-coated with a 2.5 μm-thick RZJ-390PG-50 photoresist layer (Ruihong Electronic Chemical, China) at 3,000 rpm and soft-baked at 100°C for 2 min. After the substrate is exposed with an i-line mask aligner (ABM, USA) at a dose of 300 mJ/cm^2^, it is developed for 35 s (Ruihong Electronic Chemical, China). A layer of ZnO with thickness of about 1 nm is sputtered into a predefined opening with a diameter of 5 mm on the substrate by VTC 300 (Microtech, China) as seeds for growing ZnO nanopatterns. Subsequently, ZnO nanopatterns are synthesized by hydrothermal growth in an aqueous solution with 35 mM Zn(NO_3_)_2_•6H_2_O (Sigma, USA) and 35 mM hexamethylenetetramine (C_6_H_12_N_4_) (Sigma, USA) at 90°C for 2.5 h. After the nanopatterns are synthesized, the substrate is rinsed with deionized water. The SEM image of ZnO nanopatterns by the hydrothermal growth is shown in Figure 2D. Sputtering and lift-off processes are used to prepare Cr/Au electrodes, leads and pad patterns. Figure 2E shows SEM image of metal-coated nanopatterns. In order to prepare the insulating layer, the substrate is spin-coated with 2 μm-thick SU-8 2002 (Kayaku Advanced Materials, USA) and soft-baked at 95°C for 1 min. The effective electrode is the intersection (20 × 20 μm^2^ square) between the 20 μm-wide lead wire and the 20 μm-wide annular opening of the insulating layer, which is used as a recording site (red box in Figure 2G) to detect the cardiomyocyte signals. The i-line mask aligner is used to expose the SU-8 layer (blue in Figure 2F) at a dose of 120 mJ/cm^2^, and then develop it in propylene glycol methyl ether acetate (PGMEA) for 1 min after baking at 95°C. The device is rinsed in isopropanol, dried with a N_2_ air gun, and baked at 150°C for 1 h to complete the preparation. Afterwards, it is assembled and fixed to a customized printed circuit board (PCB) base. The device is connected to the matching pad on the PCB with conductive silver glue (Electrolube, USA), and then a glass ring is glued to the center of the device using polydimethylsiloxane (PDMS) as a cell culture well. Finally, pin headers are soldered on the PCB base for connecting the signal processing module and the electroporation module. The assembled sensor is shown in the Figure 2C.

### 2.3 Integrated System Design

**FIGURE 3:**
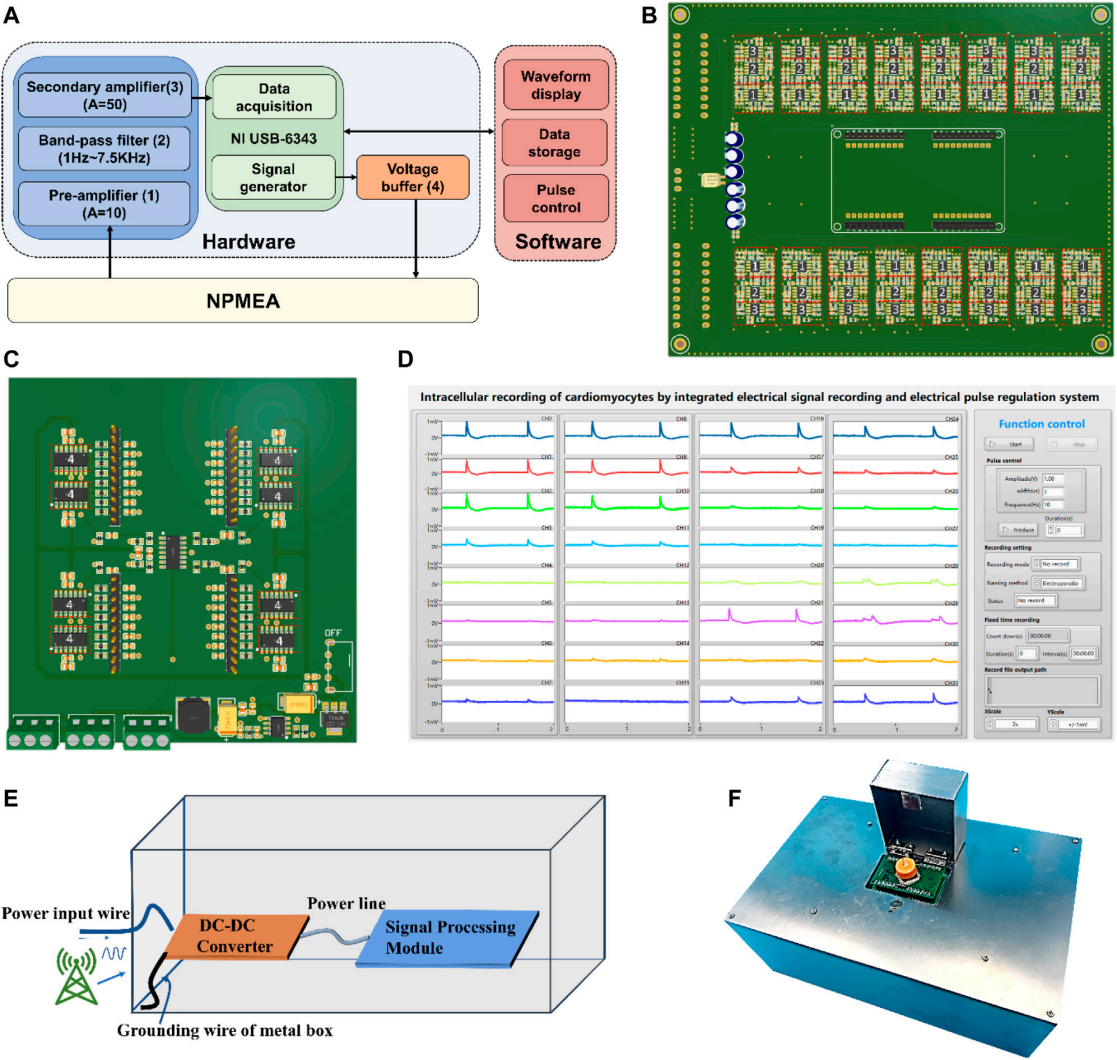
Design of integrated electric signal recording and electric pulse regulating system. **(A)**The system structure of integrated electric signal recording and electric pulse regulating system: biosensor units, hardware module, and software module. The hardware module includes a signal processing module (blue), electroporation module (orange), and a DAQ module (green). The functions of the software module include waveform display, data storage and pulse control (pink). (Numbers in parentheses indicate the corresponding labelled components in **(B,C)**) **(B)** Signal processing module (integrated 32-channel signal processing circuit) diagram. **(C)** Electroporation module (integrated 32-channel electroporation circuit) diagram. **(D)** The software user interface of integrated electrical signal recording and electrical pulse regulating system. **(E)** The internal wiring of the shielding box. The ground wire of the shielding box is connected to the ground of the power supply, which can effectively shield the interference of power frequency noise and high frequency noise. **(F)** The photo of the integrated electrical signal recording and electrical pulse regulating system.

The integrated electrical signal recording and electrical pulse regulating system consists of three modules: biosensor units, a hardware module, and a software module. The block diagram of the system is illustrated in Figure 3A. The hardware module contains a signal processing module, an electroporation module, and a data acquisition card (DAQ) module (Model USB-6343 from National Instruments Inc.). Due to the low amplitude of the cell electrophysiological signals obtained by nanopatterned electrodes and a large amount of noise introduced during the transmission, the electrophysiological signals will be attenuated and distorted. To convert the electrophysiological signals of cardiomyocytes into high-quality signals for collection, the electrophysiological signals need to be amplified and filtered through a signal processing module (Figure 3B). According to the Eq. 1, the larger the input impedance of the amplifier, the higher the coupling efficiency. A high input impedance operational amplifier is used to amplify (Gain = 10) the electrical signal, so as to minimize the attenuation of the input signal. To achieve 1 Hz-7.5 kHz band-pass filter, a resistance-capacitance (RC) high-pass passive filter (with a cut-off of 1 Hz) is used to attenuate the DC component in the signal and a Butterworth low-pass filter (with a cut-off frequency of 7.5 kHz) to eliminate high frequency noise. After amplifying the signal through a secondary amplifier (Gain = 50), it is recorded by the analog-to-digital converter (ADC) (voltage detection range: ±5 V) of DAQ. This system can sample 32-channel signals simultaneously at 15 kHz in real-time. For the recording of intracellular electrical signals, electroporation of cardiomyocytes is required. The pulse signals provided by the digital-to-analog converter (DAC) of DAQ are applied to the nanopatterned electrodes through the electroporation module (Figure 3C). In order to enhance the driving ability of the pulse and prevent the mutual influence between the channels, a voltage follower is placed in each channel. Even though there are no cells attached to the microelectrodes on some channels, it will not weaken the electroporation current on other channels. For channels with cell adhesion, the pulse current flows through the working electrodes to act on the cell, and finally flows to the reference electrode to form a loop. The impedance of the electrode branch with cell adhesion is much larger than that of electrode branch without cells. According to the shunt principle of the parallel circuit, if the channels are not isolated, most of the current in the pulse flows to the working electrodes with low impedance, resulting in smaller current in the branch with high impedance. At this time, most of the pulse current does not apply to the cell, which seriously affects the electroporation efficiency of the cell. The software based on LabVIEW is developed to control DAQ, display and save the collected electrical signals, and generate pulses. As shown in Figure 3D and Supplementary Figure S1, the software interface is divided into two areas: the function control area and the signal display area. In the measurement, the electrical signal curve can be plotted in real time.

To shield the interference of power-frequency noise and high-frequency noise, the circuit is installed in a metal shielded box. The internal wiring of the shielding box is shown in the Figure 3E. This system uses a 12 V adapter to supply power, and a DC-DC converter are then applied to convert 12 V to ±5 V as power supply for the signal processing module. The shielding box is connected to the ground wire of the power supply. The noise is directly conducted to the power supply through the power line, and will not crosstalk to the signal conditioning circuit. Therefore, the anti-interference ability of the system can be effectively enhanced. The system also uses a flip-type metal box to protect the biosensor, and the holes on the left and right sides of the metal box help cells to breathe. The assembled integrated system is shown in the Figure 3F.

### 2.4 Primary Rat Cardiomyocyte Culture

After disinfecting the device with 75% ethanol, the device is put in a biological safety cabinet and irradiated with ultraviolet light for 2 hours. In order to improve electrode compatibility and promote cell adhesion, the device is coated with 50 μl 10 ng/ml fibronectin solution (Sigma, USA) free of Ca^2+^ and Mg^2+^, and placed in a 4°C refrigerator overnight. For cell culture, Sprague-Dawley rats cardiomyocytes cells are employed. Ventricular tissue obtained from rats after surgery is rinsed in Dulbecco modified Eagle medium (DMEM) (Thermo Fisher Scientific, USA) and then cut into 1–2 mm^3^ pieces in Hanks’ Balanced Salt Solution (HBSS) (Thermo Fisher Scientific, USA). The cardiac tissue is digested with trypsin/collagenase mixture 10 to 12 times, each digestion last for 8 min, and the cell suspension is then collected and centrifuged. By twice 45-min differential adhesion, cells are derived and cultured onto the device at a density of 3 × 10^5^ cells/cm^2^ and maintained at 37°C in an incubator with 5% CO_2_.

### 2.5 Scanning Electron Microscope Sample Preparation

To prepare cell samples for scanning electron microscope, the cardiomyocytes cultured on the nanopatterned electrodes are treated with 2.5% glutaraldehyde solution for 4 h and rinsed twice with deionized water. Increasing concentrations of ethanol (30, 50, 70, 80, 90, 95, 100, 100%) are then used and critical point drying (CPD) (Samdri-795, Tousimis Research Corp.)

### 2.6 Signal Processing and Statistical Analysis

The signal processing is performed with customized MATLAB software. In the performance test of the instrument, the amplitude of the signal can be obtained by performing FFT operation on the acquisition result. The SNR is calculated as

$$SNR\left(dB\right)=20\;\times \;log10\frac{V_{Signal}}{{\text{V}}_{\text{Noise}}}$$ (2)

Where ${\text{V}}_{\text{Signal}}$ is the amplitude of signal, ${\text{V}}_{\text{Noise}}$ is the amplitude of noise. All statistical analyses are evaluated using Prism 8.0 (GraphPad Software Company, USA) or Office Excel 2016 (Microsoft, USA). All results and error bars are presented as mean ± SD (standard deviation). The data are performed with an unpaired t-test, and the difference between groups is considered to be statistically significant when *p* < 0.05.

## 3 Results and Discussion

### 3.1 Performance Testing of Integrated System

**FIGURE 4:**
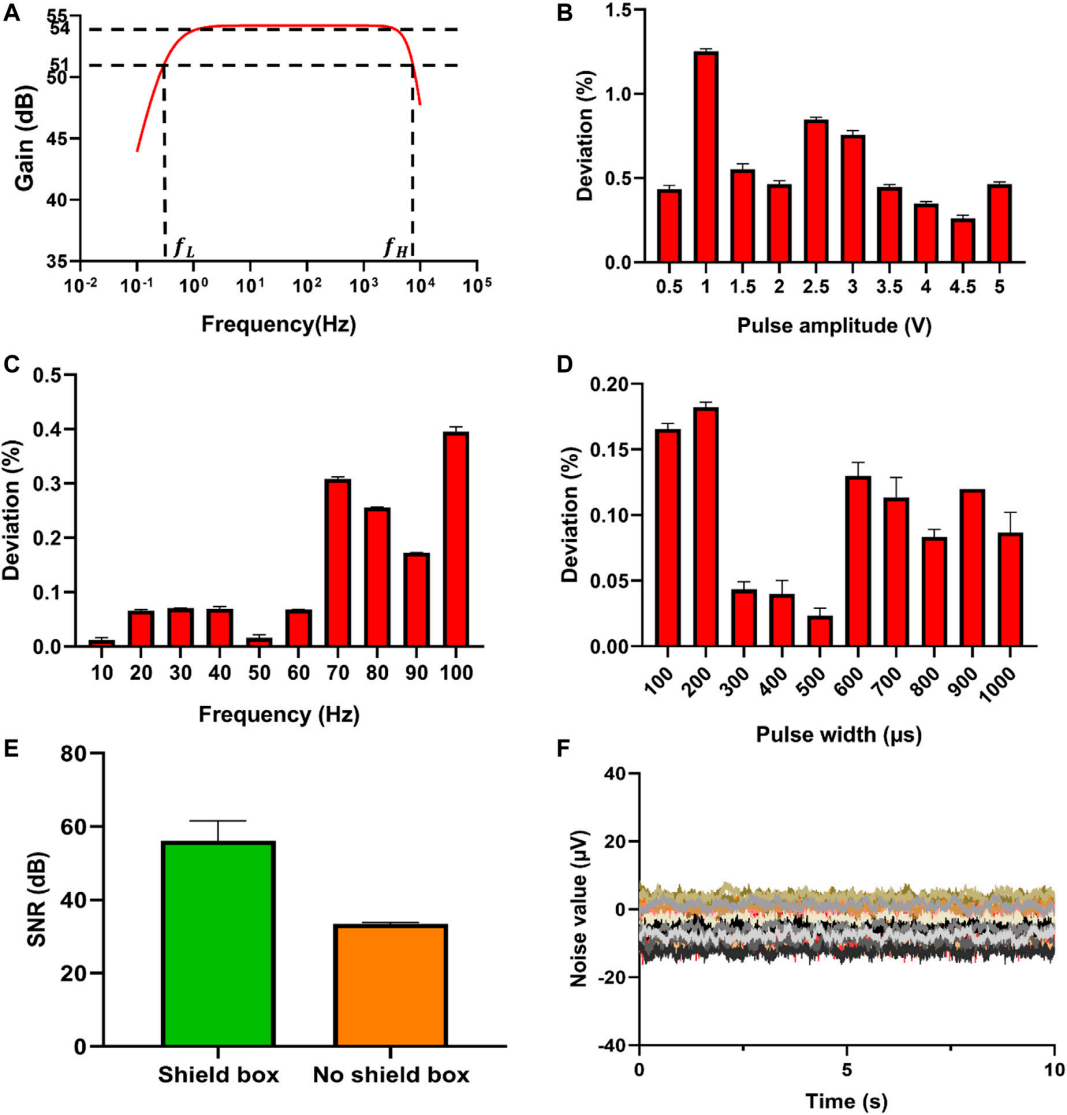
Performance test of the electrical signal recording and electrical pulse regulating system. **(A)** Bode plot of the signal processing circuit. **(B)** Deviation of the pulse amplitude generated by DAQ. **(C)** Deviation of the pulse frequency generated by DAQ. In actual experiments, the frequency range of the electroporation pulses is 10–100 Hz, so the commonly used frequency points are selected for testing. **(D)** Deviation of pulse width generated by DAQ. **(E)** SNR comparison between shield box and no shield box. **(F)** Sensor performance test of NPMEA with PBS (Curves in different colors represented all 32 channels of a sensor).

The performance of the integrated system is very important for the determination and further analysis of the electrical signal. To verify the signal processing circuit, a waveform generator DG 1032Z (RIGOL, China) is used to generate a 16 mV peak-to-peak sine wave, sweeping from 0.1 Hz to 10 MHz. The oscilloscope MS05104 (RIGOL, China) is used to detect the gain of the signal processing module at different frequencies, in order to obtain the bandwidth of the signal processing circuit. The Figure 4A shows that the passband is relatively flat with a gain of 54 dB (A = 500), and the two frequency points with a gain of 51 dB (A = 355) are 0.3 Hz and 7.4 kHz, respectively. Since the signal processing module is powered by ±5 V and the operational amplifier used at the same time is not rail-to-rail, the voltage amplitude output by the operational amplifier cannot reach ±5 V. The magnification of the signal processing module is 500 times, so the signal processing module can amplify the signals within ±10 mV. In actual measurement, intracellular signals are usually below ±5 mV due to the low sealing impedance, so our ADC range should be suitable to measure the intracellular signals. The test of the electroporation module includes pulse rising edge, falling edge, pulse width range, amplitude range, frequency range and overshoot. To measure these parameters, we use the oscilloscope instead of our data acquisition card for measurement. The sampling rate of the oscilloscope is up to 8 GHz/s. The pulse signal is the key to realize the detection of intracellular electrical signals, and electroporation requires DAQ to provide accurate pulse signals. Using the software to control DAQ to generate the corresponding pulses, and using the oscilloscope to detect DAQ output. The errors between the output of DAQ and the parameters set by the software are shown in Figures 4B–D. The amplitude deviation of the excitation signal is less than 1.5%, the frequency deviation is less than 0.15%, and the pulse width deviation is less than 0.3%, which meets the experimental requirements. It is also necessary to test properties of the electroporation circuit, including some specific parameters of the pulse. The test method for the rising edge of the pulse is to output a positive pulse with an amplitude of 4 V, and measure the time is defined from 10% (0.4 V) to 90% (3.6 V). The test method for the falling edge of the pulse is to output a positive pulse with an amplitude of 4 V, and the falling time is defined from 90% (3.6 V) to 10% (0.4 V). In addition, the impact of the metal shielding box on the circuit performance is compared (Figure 4E), and the test shows that the metal shielding box can shield the influence of external noise, thereby reducing the baseline noise of the system. Finally, using phosphate buffer solution (PBS, pH 7.4) to test the sensor performance (Figure 4F), and the peak-to-peak noise is about 30 μV. The parameters of the system are shown in the Table 1.

**TABLE 1:** Specifications of the electrical signal recording and electrical pulse regulating system.

Module	Features
Signal processing circuit	
Base noise	±15 μV
Detection voltage range	−8∼8 mV
Bandwidth	0.3∼7.4 kHz
Electroporation circuit	
Rising edge of pulse	1.018 μs
Falling edge of pulse	1.024 μs
Pulse amplitude	0∼5 V
Pulse width range	2 μs∼1 s
Pulse frequency range	1∼100 kHz

### 3.2 Electrophysiology Recording and Electroporating

**FIGURE 5:**
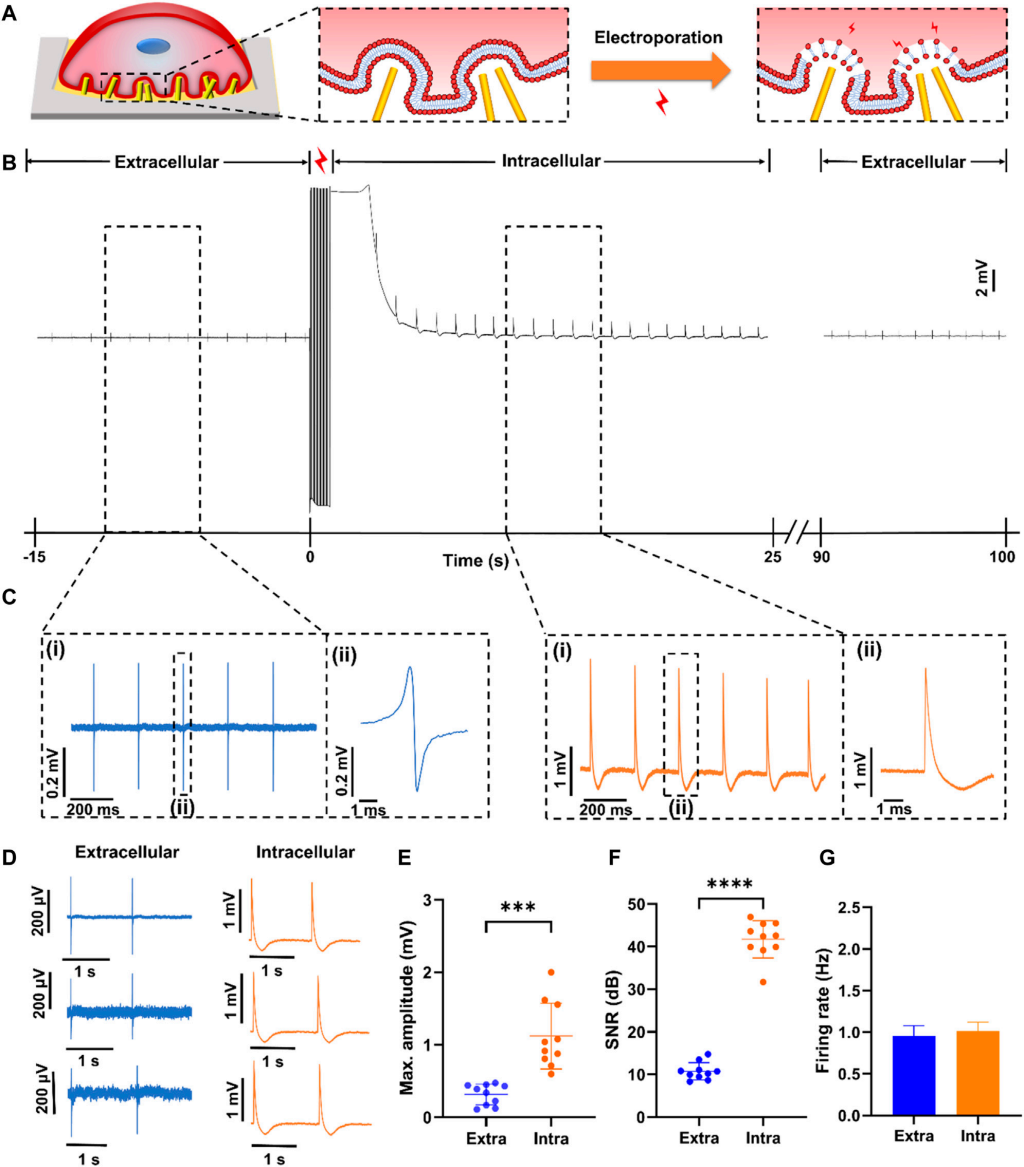
Electrophysiological signals of rat cardiomyocytes recorded by NPMEA. **(A)** Schematic diagram showing the concept of multi-site electroporation at the three-dimensional ZnO nanopatterned biocompatible interface. **(B)** Transition from extracellular measurement of cardiomyocytes to intracellular measurement with monophasic pluses (amplitude of 3 V, pulse width of 200 μs, frequency of 20 Hz, duration of 1 s) (at 0 s). After electroporation, the recorded intracellular potential amplitude decays over time due to sealing of transient pores in the cell membrane. Gradually the recording signal is restored to the shape of the extracellular signal. **(C)** The electrical signals recording before and after electroporation. (i) Partial enlarged signals in black dash box of **(B)**. (ii) Close-up view of dashed region in (ii) showing a typical signal. **(D)**Typical extracellular measurement **(left)** and intracellular measurement **(right)** of cardiomyocytes. **(E)** Plot of max amplitude of top ten intracellular and extracellular spikes. **(F)** Plot of SNR of top ten intracellular and extracellular spikes. **(G)** Plot of the firing rate of intracellular and extracellular signals.

Before signal recording, primary neonatal rat cardiomyocytes are cultured on NPMEA for 3 or 4 days. When the cardiomyocytes are located on the nanopatterned area and formed a tightly coupled biological interface with the tips and gaps of the nanopatterned electrodes (Figure 5A), the spontaneous extracellular action potential of the cardiomyocytes can be recorded by the nanopatterned electrodes with a low amplitude of about 100–500 μV, which can be observed steadily in software. After setting the pulse voltage, pulse width, frequency and other parameters of electroporation, the software will generate a single-period pulse array according to the voltage, pulse width, frequency and other parameters set by the user, and send the array to the analog output buffer of the DAQ. When electroporation occurs, the data acquisition card will output pulse waveforms for cardiomyocyte electroporation according to the set duration. Click “Produce” on the software to start electroporation. Electroporation uses rectangular pulses, which is commonly used electroporation pulse. Based on our experience, pulses with an amplitude of 3 V, a frequency of 20 Hz, a pulse width of 200 μs and a duration of 1s are applied to the nanopatterned electrodes to obtain a high-amplitude (∼0.5–5 mV) intracellular action potential (D. Xu, Fang, et al., 2021). Figure 5B shows a recording before and after electroporation. After electroporation, the system can quickly switch from stimulation to recording mode on the same electrode. When electroporation is applied to make the cells in a transient high electric field environment to form tiny nanopores at the junction of the cell membrane and the nanopatterned electrodes, which makes the signal recording change from the extracellular potential to the intracellular potential. Figure 5C shows the partially enlarged signal in Figure 5B. Unlike extracellular potential, the intracellular signal presents high amplitude. The sharpness of the action potential indicates that the nanopatterned electrodes successfully penetrated the cell. When the nanopatterned electrodes enter the intracellular environment through electroporation, the signal amplitude significantly rises. With the cell membrane resealing, the intracellular electrical signal gradually returns to the extracellular electrical signal. It can be seen from the Figure 5G that the frequency and amplitude of extracellular signal after electroporation are basically consistent with that before electroporation, indicating the biosafety of electroporation. Because we use nanopatterned electrodes, dense electrodes will produce electroporation at multiple points, but not all electrodes will enter the cell. Electrodes that have not entered the cell may enter the cell after the next electroporation. As the cell membrane is resealed, some pillars on the same electrode remain inside the cell, and some pillars become outside the cell as the cell membrane is sealed. At this time, the signal recorded by the electrode will appear as a synchronized signal inside and outside the cell. At the same time, some electrodes remain inside the cell, while other electrodes are outside the cell. At present, we only used the sensor to verify the overall function of the system and used a single condition of electroporation. The results show that the system can detect intracellular signals in parallel (Figure 5D). 87% perforation efficiency refers to the ratio of successful intracellular recording channels to the total channels after electroporation. Some channels still present combined intracellular and extracellular signal.

### 3.3 Performance of Intracellular Recording

After electroporation, the frequency of the intracellular electrical signal is basically the same as that of the extracellular electrical signal, but the quality of the intracellular signal has been greatly improved, which is mainly reflected in the amplitude and SNR. Compared with the amplitude of the extracellular electrical signal (0.315 ± 0.144 mV), the amplitude of the intracellular electrical signal (1.12 ± 0.454 mV) increases significantly (Figure 5E). The amplitude of the intracellular electrical signal is not only related to the coupling between the cell and the electrode, but also related to the effect of electroporation (Wagenaar et al., 2004). The pulse amplitude and duration determine the effect of electroporation. This system can flexibly set the electroporation conditions, and in subsequent experiments, it can be explored under which conditions the cardiomyocytes are most efficiently electroporated. In addition, the SNR of intracellular electrical signals (up to 46 dB) is greater than that of extracellular electrical signals (Figure 5F). From the analysis of amplitude and SNR, using nanopatterned electrodes combined with electroporation technology to record intracellular electrical signals can improve the signal quality. It also verified that the nanopatterned electrodes have good structural stability and can form a tight coupling with cardiomyocytes.

## 4 Conclusion and Perspectives

In this work, we developed an integrated electrical signal recording and electrical pulse regulating system for the recording of intracellular signals with NPMEA. The system can simultaneously sample multichannel signals in real time. At the same time, the system can flexibly set electroporation conditions. Compared with using a function generator as an electroporation pulse source, the system can not only accurately control the duration of electroporation, but also improve the efficiency of electroporation for the cardiac electrophysiology.

Although this work has achieved initial success in intracellular recording, there are many areas that need to be improved. For example: 1) To allow the integrated system to be tested in the cell incubator for a long time, a separate instrument can be designed to reduce the heating of the instrument by simplifying the circuit put in the cell incubator. 2) In order to simplify the experimental process of finding the best perforation parameters, an automatic optimal electroporation condition analysis and screening function can be developed to control each channel to produce different electroporation conditions. In this way, the optimal electroporation conditions can be screened in one experiment, avoiding the waste of experimental samples. Meanwhile, high-efficiency automated analysis can be realized through the software, and the feature points of intracellular signals can be automatically extracted using signal feature extraction algorithms. After screening the optimal perforation pulse parameters, cell electroporation can be performed under optimal conditions to achieve high-quality intracellular recording of cardiomyocytes. With the continuous advancement of science and technology, the integrated system with recording and stimulation functions will develop towards high throughput, high quality and intelligence, providing a reliable detection platform for the growing demand of cell electrophysiological experiments.

## Data Availability

The original contributions presented in the study are included in the article/Supplementary Material, further inquiries can be directed to the corresponding authors.
