# Supplementary material for: Intracellular Recording of Cardiomyocytes by Integrated Electrical Signal Recording and Electrical Pulse Regulating System
Source: Front Bioeng Biotechnol. 2021 Dec 15;9:799312. doi: 10.3389/fbioe.2021.799312 (PMC8714743; doi:10.3389/fbioe.2021.799312)
Supplement: Supplementary file 1 [file Image1.pdf]

Supplementary Material for

1 The detailed information of software.

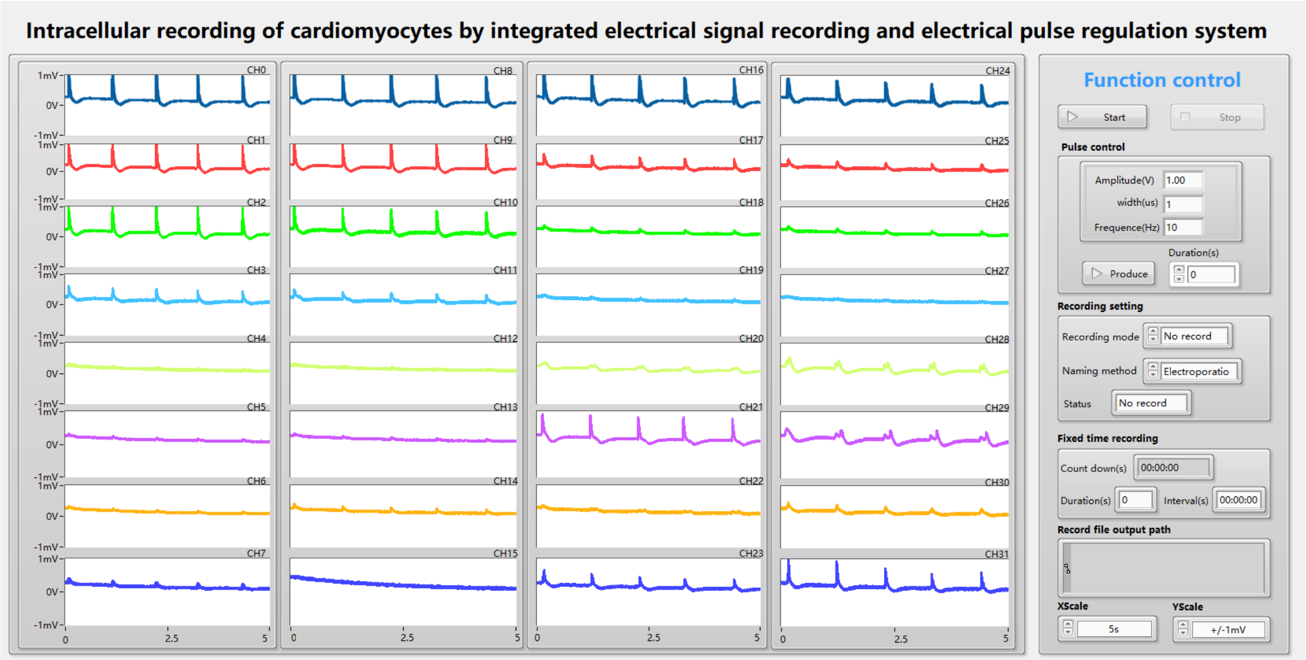

Supplementary Figure 1. The software user interface of integrated electrical signal recording and electrical pulse regulating system. The function control area mainly includes 'Start', 'Stop', 'Pulse control', 'Recording mode', 'Record file output path', and 'Waveform adjustment'. The 'Start' and 'Stop' buttons are used to control the start and stop of the signal recording. 'Pulse control' is used to control pulse parameters, including pulse amplitude, frequency, pulse width and duration. 'Recording mode' is divided into three modes: no record, continuous record, and fixed time record. 'Record file output path' is the path where the data file (TDMS) is saved. 'Waveform adjustment' is used to select the X-axis time length and Y-axis amplitude range of the waveform. The left side of the software interface is the signal display area, with 32 signal waveforms. In the measurement, the electrical signal curve can be plotted in real time.
